# Systemic inflammation in a melanoma patient treated with immune checkpoint inhibitors—an autopsy study

**DOI:** 10.1186/s40425-016-0117-1

**Published:** 2016-03-15

**Authors:** Viktor H. Koelzer, Sacha I. Rothschild, Deborah Zihler, Andreas Wicki, Berenika Willi, Niels Willi, Michèle Voegeli, Gieri Cathomas, Alfred Zippelius, Kirsten D. Mertz

**Affiliations:** Institute of Pathology, Cantonal Hospital Baselland, Mühlemattstrasse 11, CH-4410 Liestal, Switzerland; Translational Research Unit (TRU), Institute of Pathology, University of Bern, Murtenstrasse 31, CH-3010 Bern, Switzerland; Division of Medical Oncology, University Hospital Basel, Petersgraben 4, CH-4031 Basel, Switzerland; Department of Medical Oncology, Cantonal Hospital Baselland, Mühlemattstrasse 11, CH-4410 Liestal, Switzerland; Institute of Radiology, Cantonal Hospital Baselland, Mühlemattstrasse 11, CH-4410 Liestal, Switzerland

**Keywords:** Melanoma, Immunotherapy, Immune checkpoint inhibitors, Antibody, Ipilimumab, Nivolumab, Autoimmunity, Autopsy, Anti-tumor T cell response

## Abstract

**Background:**

Immune checkpoint inhibitors targeting cytotoxic T-lymphocyte-associated protein 4 (CTLA-4) and programmed cell death protein 1 (PD-1) have been recently approved for treatment of patients with metastatic melanoma and non-small cell lung cancer (NSCLC). Despite important clinical benefits, these therapies are associated with a diverse spectrum of immune-related adverse events (irAEs) that are typically transient, but occasionally severe or even fatal.

**Case presentation:**

This autopsy case illustrates that clinically overt irAEs may represent only a fraction of the total spectrum of immune-related organ pathology in patients treated with immune checkpoint inhibitors. We report a comprehensive analysis of systemic irAE pathology based on the autopsy of a 35-year-old female patient with metastatic melanoma treated first with ipilimumab and then nivolumab. The clinical course was characterized by a mixed tumor response with regression of skin and lung metastases and fatal progression of metastatic disease in the small bowel, peritoneum and brain. During therapy with ipilimumab, radiographic features of immune-related pneumonitis were noted. The autopsy examination established a sarcoid-like granulomatous reaction of the lung, pulmonary fibrosis and diffuse alveolar damage. Importantly, a clinically unapparent but histologically striking systemic inflammation involving the heart, central nervous system, liver and bone marrow was identified. Severe immune-related end-organ damage due to lymphocytic myocarditis was found.

**Conclusions:**

Autopsy studies are an important measure of quality control and may identify clinically unapparent irAEs in patients treated with immunotherapy. Pathologists and clinicians need to be aware of the broad spectrum of irAEs for timely management of treatment-related morbidity.

**Electronic supplementary material:**

The online version of this article (doi:10.1186/s40425-016-0117-1) contains supplementary material, which is available to authorized users.

## Background

Four years after the approval of the first checkpoint inhibitor ipilimumab (anti-CTLA-4) for advanced melanoma in 2011, cancer immunotherapy is now considered one of the pillars of cancer therapy [1]. Immune checkpoint inhibitors interacting with the PD-1/PD-L1 axis were recently approved by the Food and Drug Administration (FDA) based on successful large randomized controlled clinical trials [2] of patients with metastatic melanoma [3, 4], non-small cell lung cancer (NSCLC) [5, 6] and renal cell cancer [7]. There is a broad activity in different cancer types including DNA mismatch repair deficient colorectal cancer [8], ovarian cancer [9] and treatment-refractory Hodgkin lymphoma [10]. Durable responses with survival plateaus have been reported. As a consequence, the number of patients treated with immunotherapy is expected to increase. Both pathologists and clinicians therefore need to be increasingly aware of the unique spectrum of tissue reactions associated with immune checkpoint inhibitor therapy to guide patient management in daily practice.

**Fig. 1:**
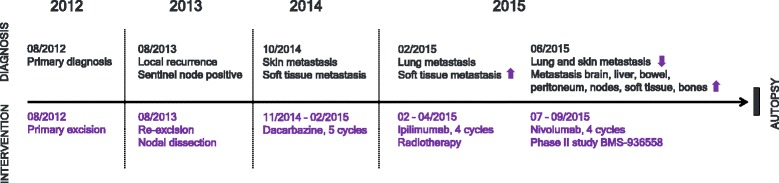
Time axis. Line graph illustrating disease progression and therapeutic intervention between initial diagnosis in August 2012 and death from metastatic melanoma in September 2015

Efficacious cancer treatment with checkpoint inhibitors can cause systemic immune activation that may potentially lead to tissue damage. Common adverse reactions affect the skin, gastrointestinal tract, liver, endocrine organs and lungs, ranging from clinically unapparent to severe immune-mediated organ damage [11]. The severity of irAEs clearly correlates with the dose and length of anti-CTLA-4 and anti-PD-1 treatment [12]. In particular, combination therapy with several immune checkpoint inhibitors may cause more adverse drug reactions than monotherapy [13]. Interestingly, a weak correlation of the severity of irAEs with treatment response has also been described [14]. Consequently, irAEs may be more common in long term survivors. Several case reports have previously illustrated the diverse clinical spectrum of irAEs including diffuse alveolar damage and immune mediated pneumonitis [15], myocarditis [16], arthritis [17], severe skin toxicity [11], hypophysitis and meningoencephalitis [18]. Due to the strong immune activation by checkpoint inhibition, it may be assumed that less severe adverse drug reactions accompany overt irAEs in patients treated with immunomodulators and may contribute to long term treatment-related organ damage. Even though analyses of systemic organ pathologies based on autopsy studies following treatment with immune checkpoint inhibitors are an important measure of quality control, postmortem studies are currently lacking in the literature. Here we report a comprehensive analysis of systemic irAE pathology based on the autopsy of a 35-year-old female patient with metastatic melanoma sequentially treated with ipilimumab and nivolumab (Fig. 1).

## Case presentation

**Fig. 2:**
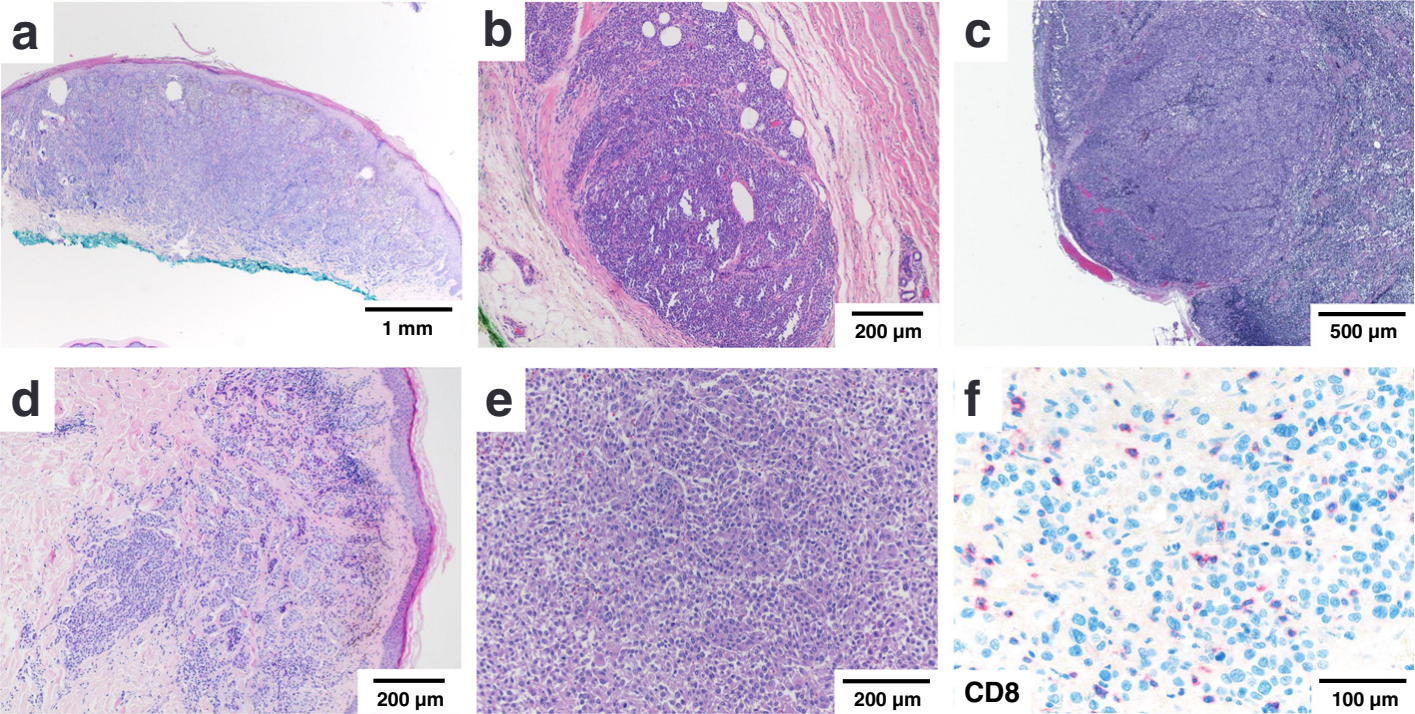
Morphological progression from initial diagnosis. **a** Melanoma ex naevo (08/2012) **b** local recurrence (08/2013) with deep in transit metastasis and **c** sentinel node metastasis **d** skin metastasis (10/2014) **e** dedifferentiated melanoma at autopsy **f** intratumoral CD8-positive T-cell infiltrates at autopsy as detected by immunohistochemistry; scale bars as indicated

In August 2012, the patient presented with a malignant melanoma arising from a congenital nevus in the right dorsum of the foot which had been diagnosed following excisional biopsy at a local primary care physician (Breslow thickness 1.7 mm, Clark Level IV) (Fig. 2a). A wide excision of the lesion with adequate safety margins was performed and the patient was lost to follow up. In August 2013, one year after the primary excision, a local recurrence of malignant melanoma was detected (diameter 1.55 mm, infiltration depth 1.55 mm). Histopathological examination revealed an in-transit metastasis (diameter 3 mm) in the subcutaneous tissue which focally reached the deep surgical margin (Fig. 2b). Re-excision with adequate safety margins and a sentinel lymph node dissection was performed, identifying melanoma micrometastases in two out of four lymph nodes examined (Fig. 2c). Following a positive pregnancy test, active surveillance was maintained.

One year after the first recurrence and 4 months after delivery, the patient presented to her dermatologist for a follow up examination. A positron-emission tomography was performed revealing enlarged and enhancing right inguinal lymph nodes with soft tissue extension. Three weeks later, multiple skin metastases on the right leg were detected and confirmed as melanoma by punch biopsy (Fig. 2d). Molecular analysis of one skin metastasis was performed at an outside institution. No potentially targetable BRAF, NRAS or c-KIT mutations were reported.

**Fig. 3:**
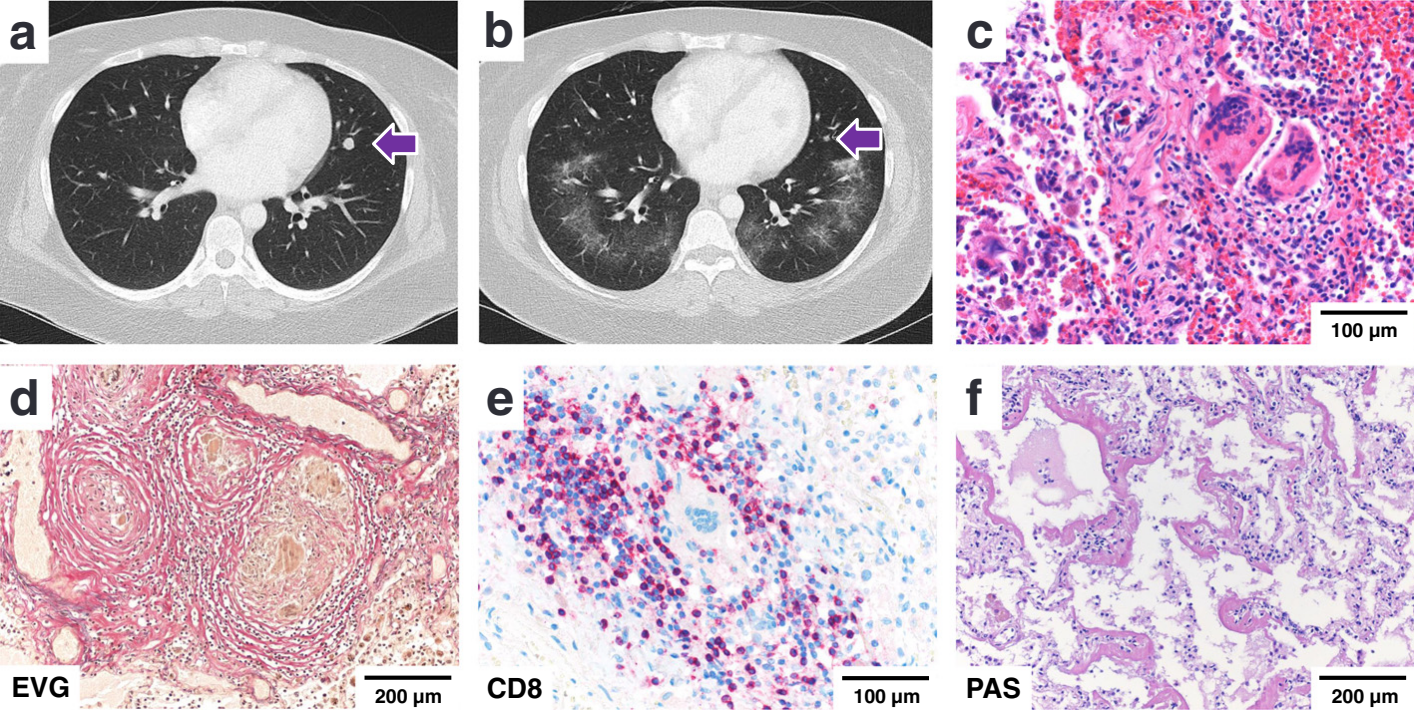
Lung damage patterns. **a** CT Thorax: Pulmonary metastasis before therapy with Ipilimumab (02/2015) **b** CT Thorax: pulmonary metastasis regression, ground glass opacities after Ipilimumab (04/2015) **c** sarcoid-like reaction **d** elastica stain showing epithelioid granulomas surrounded by fibrotic rings **e** CD8-positive T-cell infiltrates surrounding giant cell granulomas as detected by immunohistochemistry **f** diffuse alveolar damage; scale bars as indicated

First line therapy with dacarbazine every 3 weeks for 5 cycles was initiated in November 2014. The patient experienced disease progression under dacarbazine treatment with increasing size and number of skin, nodal and soft tissue metastases as well as newly detected metastases in both lungs (Fig. 3a). As dacarbazine can cause hematopoietic depression with severe leukocytopenia and thrombocytopenia, the differential blood counts were closely monitored. After the third cycle of dacarbazine, hematological studies showed significantly reduced neutrophil counts. No other hematological abnormalities were detected. Liver, renal and thyroid function was normal (see Additional file 1: Table S1 for laboratory studies).

Following completion of the fifth cycle of dacarbazine, the patient was treated with ipilimumab at 3 mg/kg every 3 weeks for four cycles from February to April of 2015. Radiotherapy to the soft tissues and nodes in the left inguinal region was administered (60Gy, 30 fractions, March to April 2015). After completing the fourth cycle of ipilimumab in the end of April 2015, computed tomography showed evidence of a mixed tumor response with regression of pulmonary and skin metastases. The appearance of bilateral pulmonary ground glass opacities was noted (Fig. 3b). The patient was closely monitored. As there was no evidence of reduced pulmonary function, no treatment was given. Differential blood counts, liver, renal and thyroid function tests were within normal range [Additional file 1: Table S1].

In June 2015, radiographic follow-up identified new metastatic lesions in the liver, the abdominal wall, the pelvic peritoneum, uterus and ovaries, spine and pelvic bones. On the first of July, 2015, Nivolumab therapy was initiated at 3 mg/kg every 2 weeks for four cycles as part of a Phase II study (BMS-936558). Repeated diffuse bleeding from metastatic lesions in the abdominal cavity and macrohematuria affecting the HB-level (Additional file 1: Table S1) required red blood cell transfusions. Multiple brain metastases were detected by computed tomography in the beginning of September of 2015. The patient suffered from intense nausea, head and neck pain and was treated with intravenous opioids and corticoids. C-reactive protein levels were elevated (250 mg/L) without fever. Laboratory values were significant for anemia, neutrophilia, lymphopenia, reactive thrombocytosis, moderately elevated aspartate aminotransferase (GOT), elevated thyroid stimulating hormone (TSH) and lactate dehydrogenase (LDH) values. Peritoneal taps were performed showing bloody ascites and elevated leucocyte counts, consistent with spontaneous bacterial peritonitis. Rapid progression of intraabdominal disease led to ileus and renal failure. The patient deceased under best supportive care. An autopsy was performed.

### Autopsy findings

Partially necrotic, amelanotic melanoma metastases were detected in brain, liver, soft tissues, small bowel, pelvic peritoneum, uterus and ovaries (Fig. 2e). Complete regression of the pulmonary metastases, osseous metastases and skin lesions was documented. A prominent intratumoral cytotoxic T-cell infiltrate with up to 100 CD8+ T-cells/mm^2^ (Fig. 2f), frequent expression of PD1 and cytotoxic granule-associated RNA binding protein (TIA-1) (Additional file 5: Figure S1) as well as a prominent histiocytic infiltrate and tumor necrosis were noted in the majority of lesions examined.

Histopathologic examination of the lungs revealed two pathogenetically distinct tissue reaction patterns. First, we observed panlobular histiocytic granulomas with giant cells (Fig. 3c), perifocal interstitial lymphocytic infiltrates and fibrotic rings (Fig. 3d) in both lungs with an interlobular, peribronchiolar, and subpleural distribution. The perifocal lymphocytic infiltrate was rich in CD8-positive T-cells (Fig. 3e) with frequent expression of TIA-1 and PD-1. Central necrosis was absent. No increase in eosinophils or mast cells was detected. Second, an acute and multifocal pattern of diffuse alveolar damage with formation of hyaline membranes was observed in all pulmonary lobes (Fig. 3f). No residual melanoma cells were detected by S100 immunohistochemistry. The patient history was non-significant for allergies or occupational exposure to dust or silica. PAS and silver stains for fungi as well as Ziehl-Neelsen stains for mycobacteria were negative. Tissue based polymerase chain reaction (PCR) analysis for mycobacteria, francisella tularensis, bartonella henselae, CMV, HSV, VZV, EBV, mucor and aspergillus were negative. Autoimmune and infectious disease serology and tissue testing was negative (see Additional file 2: Table S2).

**Fig. 4:**
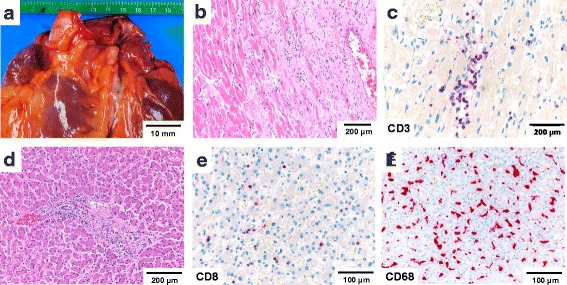
Lymphocytic myocarditis and hepatic T-cell infiltration. **a** Normal-sized heart with normal coronary arteries in frontal view **b** patchy myocardial fibrosis and mononuclear infiltrates **c** myocardial CD8-positive T-cell infiltrates as detected by immunohistochemistry **e** mild hepatic portal T-cell infiltrates **f** lobular hepatic CD8-positive T-cell infiltrates and **g** sinusoidal infiltration by CD68+ histiocytic as detected by immunohistochemistry; scale bars as indicated

Autopsy analysis of the heart demonstrated an age-appropriate coronary status with isolated early stage atheromatous plaques (Fig. 4a). Histopathological examination of the myocardium revealed patchy fibrosis and diffuse mononuclear infiltrates (Fig. 4b) with up to 45 CD3-positive lymphocytes/mm^2^ (normal range 5.3 ± 5.7/mm^2^ [19]) (Fig. 4c) and up to 18 CD68-positive macrophages/mm^2^ (normal range 9.3 ± 4.3/mm^2^ [19]). Sixty five percent of infiltrating lymphocytes expressed CD8. In CD8-positive areas, up to 85 % of T-cells were PD-1 positive, 35 % expressed TIA-1. No signs of vasculitis, no granulomas or giant cells and no viral inclusions were detected. PCR-based testing of the myocardium for bacteria, CMV, HSV, VZV and EBV was negative. The patient had no clinical history of cardiac disease. Examination of the liver demonstrated a mildly increased portal and panlobular CD8-predominant T-cell infiltration (Fig. 4d-e). Abundant sinusoidal CD68+ histiocytes with foam cell morphology and formation of loose aggregates was noted (Fig. 4f). There was no evidence of viral hepatitis, storage diseases, fatty liver disease or cholangitis. No granulomas were detected. Serology was negative for HIV, Hepatitis B and C. Screening tests for anti-nuclear antibodies and autoimmune serology was negative (see Additional file 2: Table S2). There was no history of a preexisting liver disease. A list of stains performed and staining protocols can be found in (Additional file 3: Table S3 and Additional file 4: Table S4).

**Fig. 5:**
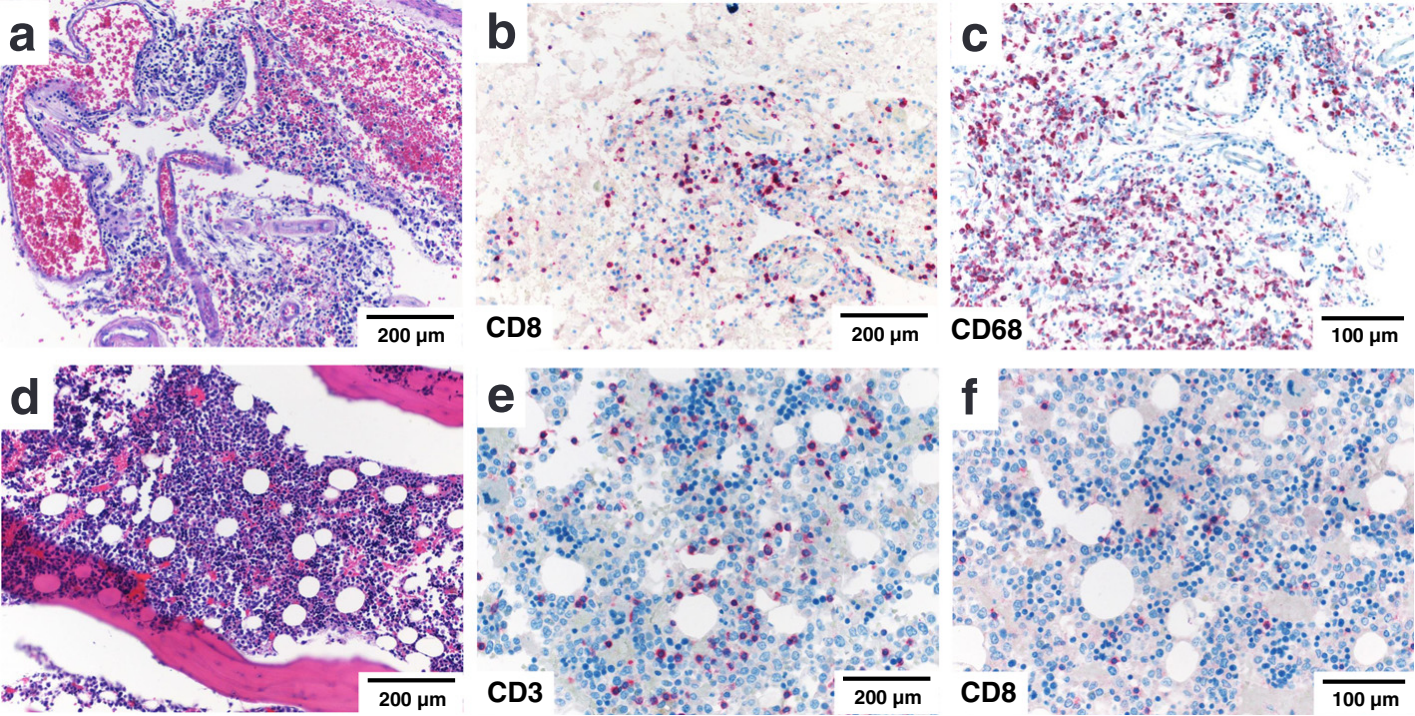
Meningoencephalitis and bone marrow lymphocytosis. **a** Meningeal lymphocytosis with **b** a predominantly CD8+ T-cell infiltrate as demonstrated by immunohistochemistry and **c** extension into the brain parenchyma; **d** Hypercellular bone marrow with **e** an increase in CD3+ and **f** CD8+ T-lymphocytes as detected by immunohistochemistry; scale bars as indicated

Histopathologic examination of the brain was pertinent for severe aseptic lymphocytic meningitis with extension of mononuclear infiltrates into the brain parenchyma (Fig. 5a). T-cell subtyping revealed that the lymphocytic infiltrate predominantly consisted of CD8+ T-cells with up to 180 CD8 + T-cells/mm^2^ detected in the meninges and up to 15 CD8+ T-cells/ mm^2^ detected in the periventricular brain parenchyma (Fig. 5b). Up to 45 % of T-cells expressed PD-1, up to 60 % showed reactivity for TIA-1 (see Additional file 5: Figure S1) with a strong concomitant CD68+ histiocytic infiltrate (Fig. 5c). There was no evidence of viral inclusions, vasculitis or ischemic lesions. The bone marrow was hypercellular with evidence of diffuse lymphocytosis (Fig. 5d). Up to 15 % of all nucleated cells in the bone marrow were CD3+ lymphocytes (Fig. 5e) and up to 10 % were CD79+ B-cells. Interestingly, only approximately 20 % of T-lymphocytes expressed CD8 (Fig. 5f). Analysis of endocrine organs including adrenals and thyroid glands did not reveal any abnormalities.

## Discussion

Immune checkpoint inhibition directed against PD-1 and CTLA-4 has the potential to activate effector T-cells against a wide spectrum of tumor- and self-antigens. The present case demonstrates the importance of systematic postmortem studies to identify relevant safety findings in this setting. We illustrate that clinically overt irAEs may be accompanied by a wide spectrum of unsuspected autoimmune pathologies that require timely treatment. Both pathologists and clinicians need to be increasingly aware of the unique spectrum of immune related adverse drug experiences for optimal patient management in daily practice.

In patients with metastatic disease, a significant fraction of tumor-antigen specific effector and memory T-cells may be detectable in the tumor and peripheral circulation, yet may be constrained by tumor-induced immune suppression mechanisms. Targeting the PD-1/PD-L1 and CTLA-4 signaling axis by immunomodulatory antibodies can induce a significant anti-tumoral immune response. However, therapeutic response to ipilimumab is accompanied by clinically detectable irAEs in up to 72 % of patients with grade 3–4 irAEs in 24 % and lethal outcome in 0.86 % of cases [11]. Onset of irAEs occurs on average 10 weeks after the onset of treatment and correlates with dosage but can occur as late as 2 years after initialization of treatment [11, 15]. In the landmark CheckMate 037 trial of nivolumab in advanced melanoma, irAEs have been observed at a similar frequency of 68 % with less common grade 3–4 events (9 %) [20]. This rate of grade 3 or 4 toxicity is similar to that seen with many chemotherapeutic agents or targeted therapies [21]. The conducted large clinical trials include a systematic assessment of irAEs under anti-CTLA-4 and anti-PD-1 blockade. However, these analyses are primarily based on clinical, laboratory and radiographic evidence with few cases reporting the analysis of tissue biopsies. Systematic autopsy studies of patients treated with immune checkpoint inhibitors are so far lacking in the literature.

This comprehensive autopsy study of a young patient treated sequentially with ipilimumab and nivolumab demonstrates that clinically or radiographically apparent organ dysfunction may represent only a small part of treatment-related unfavorable medical occurrences in a given case. In particular, a sarcoid-like pulmonary reaction, features of diffuse alveolar damage, aseptic meningoencephalitis and myocarditis with myocardial damage were discovered at autopsy as concomitant findings of a clinically suspected immune-related pneumonitis. These findings were classified as irAEs based on the close temporal associations between immune checkpoint inhibitor therapy and histopathologic findings with a significant increase of CD8+, TIA1+ and PD-1+ T-cells in the affected organs. Less significant findings included hepatic lymphocytic infiltration and bone marrow lymphocytosis.

As the patient received both ipilimumab and nivolumab, a definite association of a particular damage pattern to one of the two agents is not possible in the present case. However, the temporal association of the CT-graphic finding of ground glass opacities after ipilimumab therapy may favor an assignment of these pulmonary findings to this agent. Indeed, CTLA4-related pneumonitis during cancer immunotherapy has been previously described as a rare event, but was diagnosed based on radiographic assessment only [22]. We found a pattern of multifocal diffuse alveolar damage with hyaline membranes underlying this radiographic finding. In addition, a pulmonary sarcoid-like granulomatosis was identified. Previous CT-scans and medical history was insignificant for sarcoidosis, and post-mortem tissue based analysis ruled out an infectious etiology. Interestingly, a similar occurrence following CTLA-4 blockade has been described by other authors [11, 23]. These findings may indicate that combined checkpoint blockade may also cause superimposed histopathological damage patterns as a correlate of distinct immunological effects. Indeed, combination therapy leads to divergent gene expression changes in T-cells and monocyte populations that may underlie these specific irAEs [24]. Immune checkpoint inhibitor therapy has been linked to the uncontrolled release of cytokines in the form of a cytokine storm [25]. While the typical symptoms of a cytokine storm such as high fever, vasodilation, peripheral edema and distributive shock were not detected in the present case, cytokine release may have contributed to the development of irAEs. In particular, interleukin-2 (IL-2) secretion by activated T-cells is thought to play a role in the pathogenesis of sarcoidosis [26] and may be a mechanism linking sarcoid-like granulomatous reactions to immunotherapy. Indeed, the disease activity of a preexisting sarcoidosis may also be increased by high dose IL-2 treatment of neoplasia [27] and human immunodeficiency virus (HIV) infection [28]. Larger case series would be desirable to further investigate the mechanistic link between immune checkpoint inhibition and the development of sarcoid-like irAEs.

Cardiac autopsy findings demonstrated a lymphocytic myocarditis with patchy fibrosis in the absence of clinical, serological or tissue-based evidence for an infectious etiology. A non-infectious myocarditis in patients treated with nivolumab [3, 29] and ipilimumab [4] has been previously described, but biopsy studies are rare. Here we demonstrate a pattern of diffuse lymphocytic infiltrates with a strong predominance of CD8+/PD-1+/TIA1+ cytotoxic T-cells and concomitant diffuse CD68+ histiocytic infiltrates. These findings are reminiscent of a previous report including analysis of a myocardial biopsy from a patient treated with anti-PD-1 antibody (pembrolizumab) suggesting a similar pathogenetic mechanism [16]. It is important to note the potential pathophysiologic and clinical implications of concurrent lung and heart toxicity which may be more severe and prone for critical events than either alone.

Brain autopsy revealed a severe aseptic lymphocytic meningoencephalitis driven by CD8+/PD-1+ cytotoxic T-cells. Aseptic meningitis has been reported in a patient treated with ipilimumab [18] but histologic analysis has so far been lacking. A range of neurological and endocrine adverse events has been associated with immune checkpoint inhibitors. Most frequently, autoimmune hypophysitis and thyroidits is encountered with anti-CTLA-4 treatment [11]. In the present case, no tissue based, radiographic or laboratory evidence of hypophysis or thyroid dysfunction was present.

Other findings included a bone marrow lymphocytosis and hepatic mononuclear and histiocytic infiltrates. Hepatic toxicity and elevation of liver enzymes has been described as a mostly low-grade irAE in patients treated with ipilimumab [11] and nivolumab [20]. In a biopsy study, a pan-lobular hepatitis with prominent sinusoidal histiocytic infiltrates and central vein endothelialitis has been previously suggested as a histologic clue to ipilimumab-associated hepatitis [30]. A bone marrow lymphocytosis has not been previously identified, but may be a concomitant feature of generalized T-cell activation due to immune checkpoint blockade. In the present case, no significant hematological abnormalities in the peripheral blood counts were detected.

## Conclusion

Our results contribute to a better understanding of the atypical immune toxicity associated with checkpoint inhibition by anti-CTLA-4 and anti-PD-1 antibodies. A deeper knowledge of these immune-related adverse events and its multidisciplinary management will help to reduce morbidity and therapy interruptions. Future perspectives include the concurrent administration of antibodies targeting CTLA-4 and PD-1. Our data underline that careful monitoring is of particular importance in this setting to identify potentially harmful immune pathology.

## Consent

Written informed consent was given by the legal guardians of the patient for publication of this case report and any accompanying images. A declaration of no objection was obtained from the local ethics committee (Ethikkommission Nordwest- und Zentralschweiz (EKNZ); file designation: UBE 15–106). A copy of the written consent is available for review by the Editor-in-Chief of this journal.

## Additional files

Additional file 1: Table S1. Laboratory studies. Data on differential blood counts, renal function, liver function tests and thyroid function during each treatment cycle (dacarbazine, ipilimumab, nivolumab) are provided. (DOCX 16 kb) Additional file 2: Table S2. Autoimmune serology. Table showing the screening results of autoimmune serology including systemic antibodies, anti-neutrophil cytoplasmic antibodies (ANCA) and anti-neuronal antibodies. (DOCX 16.8 kb) Additional file 3: Table S3. Immunohistochemical stains. Systematic overview of the immunohistochemical stains performed for each organ and tumor specimen. (DOCX 21.3 kb) Additional file 4: Table S4. Antibodies and staining protocols. All stains were performed on a Leica BOND III / max autostainer platform (Leica Bioystems, Muttenz, Switzerland). Information on antibody clones, pretreatment and staining protocols is provided. (DOCX 18 kb) Additional file 5: Figure S1. Expression of cytotoxic granule-associated RNA binding protein (TIA-1) and programmed cell death protein 1 (PD-1, nivolumab). Frequent expression of PD-1 and TIA-1 in tumor infiltrating T-cells (A, B) and in lymphocytic infiltrates in the peripheral organs including the meninges (C, D); scale bars as indicated. (PPTX 4.8 mb)

## Abbreviations

ANCA: anti-neutrophil cytoplasmic antibodies
CD: cluster of differentiation
CMV: cytomegalovirus
CTLA-4: cytotoxic T-lymphocyte-associated protein 4
EBV: Epstein-Barr virus
FDA: Food and Drug Administration
HSV: herpes simplex virus
irAEs: immune-related adverse events
NSCLC: non-small cell lung cancer (NSCLC)
PCR: polymerase chain reaction
PD-1: programmed cell death protein 1
TIA-1: cytotoxic granule-associated RNA binding protein
VZV: varicella-zoster virus
