# Supplementary material for: Systemic inflammation in a melanoma patient treated with immune checkpoint inhibitors—an autopsy study
Source: J Immunother Cancer. 2016 Mar 15;4:13. doi: 10.1186/s40425-016-0117-1 (PMC4791920; doi:10.1186/s40425-016-0117-1)
Supplement: Additional file 1: Table S1. — Laboratory studies. Data on differential blood counts, renal function, liver function tests and thyroid function during each treatment cycle (dacarbazine, ipilimumab, nivolumab) are provided. (DOCX 16 kb) [file 40425_2016_117_MOESM1_ESM.docx]

**Additional file 1: Table S1**

Laboratory Studies

| **Parameter**  **(Unit; reference range)** | Before first cycle of Dacarbazine | After third cycle of Dacarbazine | After last cycle of Dacarbazine / Before First cycle of Ipilimumab | After third cycle of Ipilimumab | Four weeks after last cycle of Ipilimumab | Six weeks after last cycle of Ipilimumab | Before first cycle of Nivolumab | Start of Nivolumab therapy | After second cycle of Nivolumab | After last cycle of Nivolumab |
| --- | --- | --- | --- | --- | --- | --- | --- | --- | --- | --- |
|  | **10/27/2014** | **01/07/2015** | **02/18/2015** | **04/09/2015** | **05/29/2015** | **06/11/2015** | **06/19/2015** | **07/01/2015** | **07/27/2015** | **09/01/2015** |
| **Hemoglobin**  **(g/L; 11,5 - 16,4)** | 140 | 141 | 149 | 141 | 142 | 132 | 140 | 107 | 74 ↓ | 89 ↓ |
| **Leucocytes**  **(x 10^9^/L; 4,4 - 11,3)** | 5.3 | 3.8 ↓ | 5.0 | 5.1 | 4.9 | 7.8 | 5.65 | 4.43 | 4.3 ↓ | 13.3 ↑ |
| **Neutrophils**  **(x 10^9^/L; 2.0 - 7.0)** | 3.1 | 1.8 ↓ | 2.6 | 2.0 | 2.7 | 6.5 | 3.75 | 3.09 | 3.1 | 12.4 ↑ |
| **Lymphocytes**  **(x 10^9^/L; 1.0 - 3.0)** | 1.6 | 1.4 | 1.6 | 1.0 | 1.0 | 0.6 ↓ | 0.99 ↓ | 0.76 ↓ | 0.5 ↓ | 0.4 ↓ |
| **Thrombocytes**  **(x 10^9^/L; 150 - 300)** | 234 | 197 | 256 | 234 | 284 | 228 | 181 | 315 ↑ | 388 ↑ | 581 ↑ |
| **Creatinin**  **(µmol/L; 53 - 97)** | 71 | 74 | 69 | 61 | 69 | 51 ↓ | 61 | 63 | 50 ↓ | 50 ↓ |
| **GOT**  **(U/L; <35)** | 15 | 16 | 15 | 15 | 21 | 25 | 26 | 29 | 23 | 97 ↑ |
| **GPT**  **(U/L; <35)** | 13 | 15 | 14 | 18 | 13 | 13 | 12 | 11 | 14 | 13 |
| **LDH**  **(IU/L; <500)** | 125 | 147 | 168 | 192 | 286 | 395 | 489 | 565 ↑ | 579 ↑ | 2168 ↑ |
| **TSH**  **(IU/L; 0,3 - 4,0)** | n.d. | n.d. | 1.96 | 2.7 | 2.6 | n.d. | 2.04 | n.d. | 2.89 | 6.47 ↑ |
