# Supplementary material for: Systemic inflammation in a melanoma patient treated with immune checkpoint inhibitors—an autopsy study
Source: J Immunother Cancer. 2016 Mar 15;4:13. doi: 10.1186/s40425-016-0117-1 (PMC4791920; doi:10.1186/s40425-016-0117-1)
Supplement: Additional file 2: Table S2. — Autoimmune serology. Table showing the screening results of autoimmune serology including systemic antibodies, anti-neutrophil cytoplasmic antibodies (ANCA) and anti-neuronal antibodies. (DOCX 16.8 kb) [file 40425_2016_117_MOESM2_ESM.docx]

**Additional file 2: Table S2**

| **Analysis Type** | **Reference Range** | **Units** | **Result** |
| --- | --- | --- | --- |
|  |  |  |  |
| **Systemic antibodies** |  |  |  |
| Rheumatoid factor | <15 | IU/mL | <10 |
| ANA-Titer | <1:40 | Titer | <1:40 |
| ANA-IF pattern (Hep-2) | negative |  | negative |
| Anti-dsDNA | <10 | IU/mL | <10 |
| Anti-Histone | <1 | Units | <1 |
|  |  |  |  |
| **ANCA** |  |  |  |
| ANCA-Titer | <1:20 | Titer | <1:20 |
| ANCA-IF pattern |  | negativ |  |
| Anti-MPO (MPO-ANCA) | <5 | U/mL | <5 |
| Anti-PR3 (PR3-ANCA) | <3 | U/mL | <3 |
|  |  |  |  |
| **Anti-neuronal antibodies** | Antikörper |  |  |
| Anti-neuronal antibodies (IF-Screen) | <1:50 | Titer | <1:50 |
| Anti-Hu (ANNA-1) | negative |  | negative |
| Anti-Yo (PCA-1) | negative |  | negative |
| Anti-Ri (ANNA-2) | negative |  | negative |
| Anti-CV2 (CRMP5) | negative |  | negative |
| Anti-Ma1 (PNMA1) | negative |  | negative |
| Anti-Ma2/Ta (PNMA2) | negative |  | negative |
| Anti-Amphiphysin | negative |  | negative |

Autoimmune serology
