# Supplementary material for: Systemic inflammation in a melanoma patient treated with immune checkpoint inhibitors—an autopsy study
Source: J Immunother Cancer. 2016 Mar 15;4:13. doi: 10.1186/s40425-016-0117-1 (PMC4791920; doi:10.1186/s40425-016-0117-1)
Supplement: Additional file 3: Table S3. — Immunohistochemical stains. Systematic overview of the immunohistochemical stains performed for each organ and tumor specimen. (DOCX 21.3 kb) [file 40425_2016_117_MOESM3_ESM.docx]

**Additional file 3: Table S3**

Immunohistochemical stains

| **Antigen** | **CD3** | **CD4** | **CD8** | **PD1** | **TIA-1** | **CD68** | **S100** |
| --- | --- | --- | --- | --- | --- | --- | --- |
| **Heart** | **+** | **+** | **+** | **+** | **+** | **+** | **-** |
| **Lung** | **+** | **+** | **+** | **+** | **+** | **+** | **+** |
| **Brain** | **+** | **+** | **+** | **+** | **+** | **+** | **+** |
| **Meninges** | **+** | **+** | **+** | **+** | **+** | **+** | **+** |
| **Liver** | **+** | **+** | **+** | **+** | **+** | **+** | **-** |
| **Lymph nodes** | **+** | **+** | **+** | **+** | **+** | **+** | **+** |
| **Bone marrow** | **+** | **+** | **+** | **+** | **+** | **+** | **+** |
| **Primary tumor** | **+** | **+** | **+** | **+** | **+** | **+** | **+** |
| **Local recurrence** | **+** | **+** | **+** | **+** | **+** | **+** | **+** |
| **Sentinel nodes** | **+** | **+** | **+** | **+** | **+** | **+** | **+** |
| **Skin metastasis** | **+** | **+** | **+** | **+** | **+** | **+** | **+** |
| **Peritoneal metastasis** | **+** | **+** | **+** | **+** | **+** | **+** | **+** |
| **Brain metastasis** | **+** | **+** | **+** | **+** | **+** | **+** | **+** |
