# Supplementary material for: Systemic inflammation in a melanoma patient treated with immune checkpoint inhibitors—an autopsy study
Source: J Immunother Cancer. 2016 Mar 15;4:13. doi: 10.1186/s40425-016-0117-1 (PMC4791920; doi:10.1186/s40425-016-0117-1)
Supplement: Additional file 4: Table S4. — Antibodies and staining protocols. All stains were performed on a Leica BOND III / max autostainer platform (Leica Bioystems, Muttenz, Switzerland). Information on antibody clones, pretreatment and staining protocols is provided. (DOCX 18 kb) [file 40425_2016_117_MOESM4_ESM.docx]

**Additional file 4: Table S4**

Antibodies and staining protocols

|  | **CD3** | **CD4** | **CD8** | **PD1** | **TIA-1** | **CD68** | **S100** |
| --- | --- | --- | --- | --- | --- | --- | --- |
| **Company** | **Novocastra** | **Novocastra** | **Novocastra** | **R&D** | **Beckman** | **Dako** | **Dako** |
| **Clone** | **LN10** | **4B12** | **4B11** | **Goat IgG** | **2G9A10F5** | **PG-M1** | **polyclonal** |
| **Dilution** | **1:100** | **1:320** | **1:40** | **1:80** | **1:200** | **1:100** | **1:4000** |
| **Epitope retrieval** | **pH 9, 20min, 100C^🞆^** | **pH 9, 20min,**  **100C^🞆^** | **pH 9, 30min, 95C^🞆^** | **pH 6, 20min,**  **100C^🞆^ blocking buffer** | **Enzyme 1, 5min** | **Enzyme 1, 5min** | **pH 9, 20min**  **95C^🞆^** |
| **Autostainer protocol** | **Hmix H2 (20) 100** | **Hmix H2 (20) 100** | **Hmix H2 (30) 95** | **notH H1 (20) 100** | **notH E1 (5)** | **Hmix E1 (5)** | **Hmix H2 (20) 95** |
| **Detection system** | **Bond Polymer Refine Red Detection (Leica Biosystems)** | | | | | | |
| **Counterstain** | **Hemalum** | | | | | | |
