# Supplementary figures and images for: Systemic inflammation in a melanoma patient treated with immune checkpoint inhibitors—an autopsy study
Source: J Immunother Cancer. 2016 Mar 15;4:13. doi: 10.1186/s40425-016-0117-1 (PMC4791920; doi:10.1186/s40425-016-0117-1)

## Slide 1
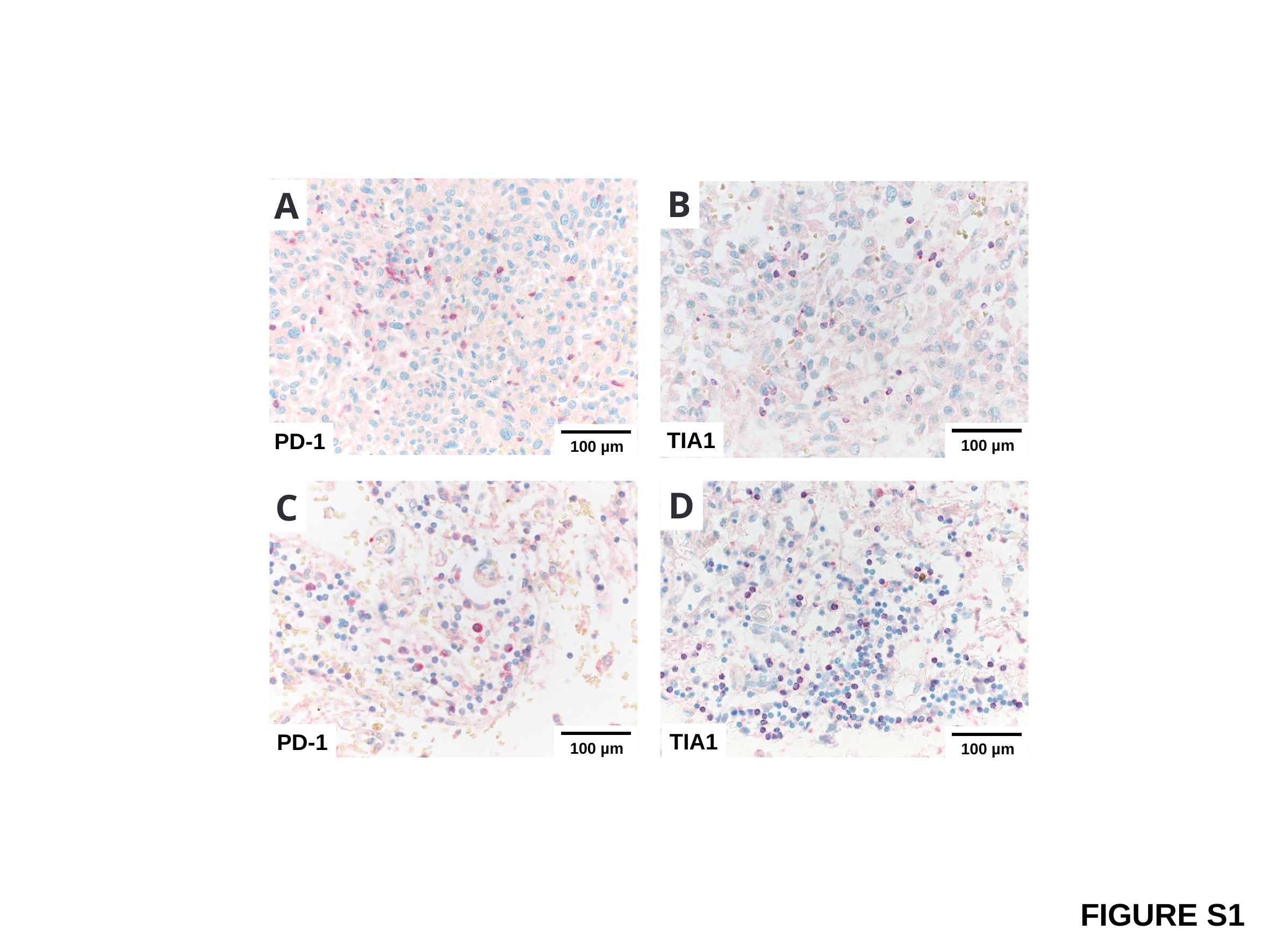

B
A
TIA1
PD-1
100 µm
100 µm
D
C
TIA1
PD-1
100 µm
100 µm
FIGURE S1

Supplement: Additional file 5: Figure S1. — Expression of cytotoxic granule-associated RNA binding protein (TIA-1) and programmed cell death protein 1 (PD-1, nivolumab). Frequent expression of PD-1 and TIA-1 in tumor infiltrating T-cells (A, B) and in lymphocytic infiltrates in the peripheral organs including the meninges (C, D); scale bars as indicated. (PPTX 4.8 mb) [file 40425_2016_117_MOESM5_ESM.pptx]
